# Neuro-musculoskeletal flexible multibody simulation yields a framework for efficient bone failure risk assessment

**DOI:** 10.1038/s41598-019-43028-6

**Published:** 2019-05-06

**Authors:** Andreas Geier, Maeruan Kebbach, Ehsan Soodmand, Christoph Woernle, Daniel Kluess, Rainer Bader

**Affiliations:** 1 Department of Orthopaedics, University Medicine Rostock, Rostock, Germany; 2 Department of Modern Mechanical Engineering, Waseda University, Tokyo, Japan; 3 Julius Wolff Institute for Biomechanics and Musculoskeletal Regeneration, Charité Berlin, Germany; 4 Chair of Technical Dynamics, University of Rostock, Rostock, Germany

**Keywords:** Computational models, Bone, Biomedical engineering

## Abstract

Fragility fractures are a major socioeconomic problem. A non-invasive, computationally-efficient method for the identification of fracture risk scenarios under the representation of neuro-musculoskeletal dynamics does not exist. We introduce a computational workflow that integrates modally-reduced, quantitative CT-based finite-element models into neuro-musculoskeletal flexible multibody simulation (NfMBS) for early bone fracture risk assessment. Our workflow quantifies the bone strength via the osteogenic stresses and strains that arise due to the physiological-like loading of the bone under the representation of patient-specific neuro-musculoskeletal dynamics. This allows for non-invasive, computationally-efficient dynamic analysis over the enormous parameter space of fracture risk scenarios, while requiring only sparse clinical data. Experimental validation on a fresh human femur specimen together with femur strength computations that were consistent with literature findings provide confidence in the workflow: The simulation of an entire squat took only 38 s CPU-time. Owing to the loss (16% cortical, 33% trabecular) of bone mineral density (BMD), the strain measure that is associated with bone fracture increased by 31.4%; and yielded an elevated risk of a femoral hip fracture. Our novel workflow could offer clinicians with decision-making guidance by enabling the first combined *in-silico* analysis tool using NfMBS and BMD measurements for optimized bone fracture risk assessment.

## Introduction

It is of major importance to reliably and rapidly obtain information on the dynamic *in-vivo* bone stresses and strains as these are fundamental parameters in mechanostat theory that characterizes the interplay between dynamic mechanical loading and musculoskeletal health^1^: Dynamic bone stresses and strains control bone remodeling^2,3^ are indicators of bone fractures^2,4,5^ and hence allow for tailored and monitored clinical treatment^6^. Additionally, dynamic stress analyses are essential to evaluate the mechanical properties of endoprosthetic implants *in-situ* during dynamic activities to improve implant design, implant durability and in turn, patient outcome^7,8^.

In this regard, bone diseases demonstrate a massive public health problem, especially bone mineral density (BMD) loss that eventually leads to fragility fractures which are associated with serious consequences in terms of morbidity, mortality, and socioeconomic burden^9^. Of the 1.7 million fragility fractures in 2011 in the US^10^ and 3.5 million fragility fractures in the E27 countries^11^, the osteoporosis-related femoral hip fracture in particular has become a major socioeconomic problem: The femoral hip fracture has a relative incidence of approximately 23%, making it the most common fragility fracture and has caused an annual treatment cost of $19 billion USD in 2004 in the US and €37 billion in 2010 in the E27. Because of the association of BMD loss with age, these numbers are expected to increase by roughly 25% until 2025 as the elderly population (>65 yrs.) continues to increase.

Targeting the early identification of adverse bone metabolism, direct measurements of stresses and strains in or on living individuals’ bones are impossible, from an ethical point of view, due to their invasiveness. Therefore, they are restricted to a limited number of superficial bone sites within cadaver studies using strain gauges^1,12–15^. In this respect, finite-element analysis (FEA) has been used extensively to address musculoskeletal research questions by means of non-invasive computational analysis; e.g., to study the mechanical behavior of human bones^13,15–18^, bone remodeling^19–21^, bone adaption^22,23^ and/or to estimate the progression of osteoarthritis in normal and overweight subjects^24–26^. Thereby, it has become a state-of-the-art method to assign the Hounsfield units (HU) of computed tomography (CT) imaging data to the finite-element mesh to capture the patient-specific bone density distribution^14,17^. This so-called quantitative CT (QCT) FEA^4,27,28^ has become a key tool in the assessment of the bone status, e.g., via predicting its mechanical bone strength^4–6,18,29,30^. For example, in assessing the risk of BMD loss-related bone fractures, it has been shown that a subject-specific FEA that utilizes QCT for material property identification, is beneficial over the current clinical standard that utilizes dual-energy X-ray absorptiometry-based (DXA) BMD measurements for diagnosis and medication effect assessment in osteoporotic patients^4,6,28–30^.

In contrast to FEA, patient-specific neuro-musculoskeletal multibody simulation (NMBS) seeks to optimize clinical decision-making by reliably predicting numerous patient-specific quantities in terms of musculoskeletal health from only scarce, non-invasive medical imaging and gait lab data^31,32^. NMBS models are regularly employed to enable rapid and reliable dynamic analysis over an enormous parameter space, to gain insight into human locomotion^33–37^ or to evaluate surgical treatment options^38^ by providing methods that quantify forces in muscles, ligaments, and joints while avoiding invasive interventions. Another application of these NMBS is the extraction of boundary conditions for FEA^23^ or the co-simulation of FEA and NMBS that can be used to investigate, for e.g., the effects of gait modifications^39,40^.

However, established clinical diagnosing standards including QCT-based FEA lack the capability to estimate stresses and strains that arise due to the dynamic multiaxial loading of the bone under varying loading conditions as usually present in daily living activities and specific to every individual^5^ to ultimately allow for the functional analysis of the bone. Moreover, FEA is usually limited to static or quasi-static loading cases and involves an immense computational cost that renders it computationally impractical for dynamic analysis of the entire neuro-musculoskeletal (NMS) system^1,23^. On the other hand, NMBS assumes bones as rigid bodies which makes the estimation of the field parameters stresses and strains impossible. In an attempt to resolve these issues, model reduction techniques have been employed within biomechanical FEA^8,41^. In the context of clinically relevant load cases that involve only small linear-elastic deformations, modally reduced flexible superelements models^42^ (SEMs) were derived from finite-element models (FEMs) of bone segments and were implemented into NMS multibody models to allow for strain computation: Hughes *et al*.^43^ observed lower tibial strains in individuals undergoing repetitive physical activity, which might suggest a lower risk of stress fractures in those individuals, due to positive bone remodeling as a result of increased physical activity. Gervais *et al*.^7^ reproduced the fracture site of an osteosynthetic plate at the lateral, distal femur in accordance with a case study and reported that only the dynamic computation of stresses yielded realistic results.

However, the implementation from SEMs into NMBS is still in its infancy, and several limitations need to be overcome to arrive at a NMBS with clinical significance. In fact, till date, an experimentally validated study investigating the translation of QCT-based FEA into the workflow of a subject-specific NMBS does not exist: It is unclear how subject-specific QCT-based FEA translates into a subject-specific NMBS in terms of the prediction performance of osteogenic field parameters and computational efficiency.

Despite these challenges, it would be desirable to enable the prediction of physiological, i.e. dynamic, bone stresses and strains during physiological tasks to monitor the bone health and provide a numerical indication for early tendencies towards abnormalities that may yield the risk of fracture. Moreover, the high prevalence of femoral hip fractures stresses the importance of monitoring dynamic stresses and strains especially in the living femur that due to the surrounding soft tissue is difficult if not impossible to analyze; thus, further complicates the validation and translation of such *in-silico* approaches into clinical practice. Therefore, the question of if and to what extent state-of-the-art NMBS can predict those biophysical stimuli for assessing the healthy and the osteoporotic femur under physiological-like conditions remains open.

## Neuro-musculoskeletal Flexible Multibody Simulation for Patient-specific Bone Failure Risk Assessment

**Figure 1:**
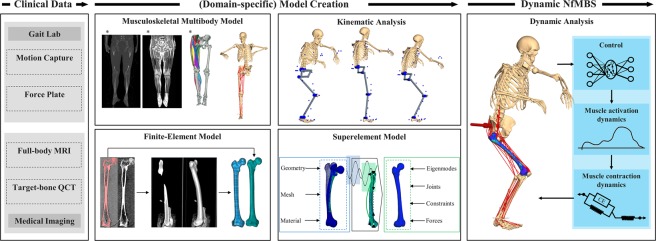
Workflow for neuro-musculoskeletal flexible multibody simulation (NfMBS) featuring dynamic stress and strain computation. The workflow is based solely on data that is typically available in clinical settings and arrives at a neuro-musculoskeletal multibody model with flexible bone segments: In conventional NMBS, motion capture and force plate data are captured in the gait laboratory, e.g., motion marker trajectories and force plate measurements that are specific to a motion scenario. Subsequently, these data are fed to computational multibody models of the neuro musculoskeletal (NMS) system that are created from medical imaging data. Depending on the specific question at hand—kinematic or dynamic—*in-silico* predictions are performed, enabling non-invasive analysis of NMS quantities. In an attempt to extend these analyses toward fragility fracture risk assessment in bones, NfMBS furthermore enables the prediction of stresses and strains that arise from the dynamic loading during clinically relevant motion scenarios by reducing QCT-based finite element models to superelement models for their integration into NMBS. The illustrations marked with * were taken from Carbone *et al*.^55^. Permission to publish is granted under a CC BY open access license.

In addressing these limitations, the objective of the present study is to augment the clinicians’ diagnosis and monitoring repertoire by enabling the reliable and rapid prediction of patient-specific stresses and strains, solely based on gait lab and medical imaging data. Hence, our work introduces a clinically motivated workflow, enabling neuro-musculoskeletal flexible multibody simulation (NfMBS) that features dynamic stress and strain computation (Fig. 1) by incorporating QCT-FEAs into conventional, subject-specific NMBS.

### Overview of the novel workflow

Our study investigates the *in-silico* prediction of BMD loss-related fracture risk in bones under the consideration of structural and functional patient specificity, i.e., of material and structural properties as well as of musculoskeletal architecture and motion dynamics of the individual NMS system.

We generated a specimen-specific FEM of a fresh human femur specimen from a male donor (age = 58 yrs) from QCT scans with an included BMD calibration phantom. Next, we derived a modally reduced flexible SEM from the very same FEM by deploying the *Craig-Bampton method*^42^. Both the domain-specific simulation models of the femur, i.e. the FEM and SEM, were validated by conducting static and dynamic biomechanical tests on the fresh human femur specimen after computer model reconstruction. Finally, two simulation studies were conducted: The first simulation study resembled the bone strength experiments done by Orwoll and coworkers^4^, in which QCT-based FEMs were deployed for the risk assessment of femoral hip fractures, due to BMD loss, within a cohort study. Then, native and BMD loss-affected SEMs were implemented into our previously presented NMBS with a computed-muscle controller^44^ featuring a full-body NMS computer model that is based on the 4^*th*^*Grand Knee Challenge to Predict In Vivo Knee Loads*^45^.

### Finite-element model validation

**Figure 2:**
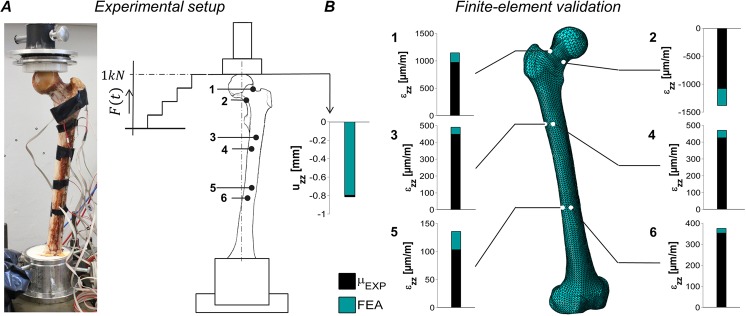
Finite-element model validation. The fresh human femur specimen was mounted onto a servo-hydraulic test machine that gradually applied a static, compressive load up to 1 kN (**A**). The vertical femoral head displacement as well as the linear strains in *z*-direction for the experimental measurements (arithmetic mean, black bar) are shown in comparison to the predictions by the finite-element analysis (FEA, green bar) (**B**).

With regard to the *bone strength* characterizing experiments, as conducted by Orwoll *et al*.^4^, we compared the experimentally measured displacement of the force application point and the (tensile/compressive) strains on the strain gauge (SG) locations SG-1 to SG-6 in response to static compressions, to the predictions of the FEA (Fig. 2).

Due to the use of linear strain gauges, the vertical strain components in *z*-direction ${{\varepsilon }}_{zz}$ alongside the femoral shaft were compared (Fig. 2B). The experimental trials allowed for reliable and repeatable strain and displacement measurements with maximum standard deviations of 9.01 µstrain among all the strain gauges and 0.0045 mm for the femoral head displacement for all the trials (confidence interval 95%, *n = *4). Note that due to technical problems with the wiring of the SG during the experiment, one out of five trials had to be discarded.

The FEM reproduced the experimental SG measurements well with an average root-mean-square-error (RMSE) of 145.25 µstrain (range 22.25 µstrain to 301.77 µstrain) between all the strain gauges and a RMSE of 0.018 mm for the vertical femoral head displacement. The computation of the FEM resembling the static experimental trial took 19.56 min CPU-time.

### Superelement model validation

**Figure 3:**
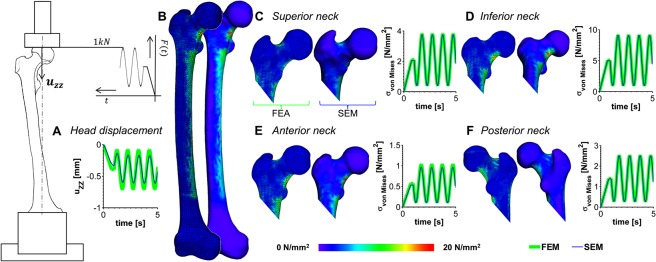
Superelement model validation against the validated finite-element model. The femoral head displacement (**A**), the *von* *Mises* stress contour plot (**B**, for the peak load of 1 kN) over the whole femur as well as in the femoral neck (**C**–**F**) as a function of time for the dynamic compression test with an amplitude of 1 kN and a frequency of 1 Hz. The predictions of the averaged (over a circular area with a radius of approx. 0.5 cm at the respective location) *von* *Mises* stresses ${{\rm{\sigma }}}_{{\rm{v}}{\rm{o}}{\rm{n}}{\rm{M}}{\rm{i}}{\rm{s}}{\rm{e}}{\rm{s}}}$ by the finite-element model (FEM, green line) are shown in comparison to the predictions by the superelement model (SEM, blue line).

As it concerns the prediction performance of the SEM in comparison to the FEM, the difference in the vertical femoral head displacement was very small with an RMSE of 0.0843 mm, over the entire simulation time. Similarly, the *von Mises* stress distribution as predicted over the whole bone surface by the SEM is in great agreement with the validated FEM. The deviation in the *von Mises* stresses computed by the FEM and the SEM were negligibly small with an average RMSE of 0.0478 MPa (range 0.0223 MPa–0.0790 MPa) over the complete simulation time (Fig. 3).

Owing to the modal reduction in the degrees of freedom (DoF) from the FEM (DoF = 370’362) to the SEM (DoF = 32), the CPU-time for the dynamic experimental trial over 5 s in real time, decreased considerably from 328.68 min for the dynamic FEA to 0.15 min for the SEM. Although the modal reduction (*t* = 13.44 min) as well as the stress and strain recovery (*t* = 14.01 min) add up to the SEM generation, the SE analysis outperformed the dynamic FEA by the factor of 11.91 in terms of CPU time. Moreover, the SEM generation is required only once; after which the SEM can be incorporated into a variety of loading cases.

### Evaluation of bone quality parameters

**Figure 4:**
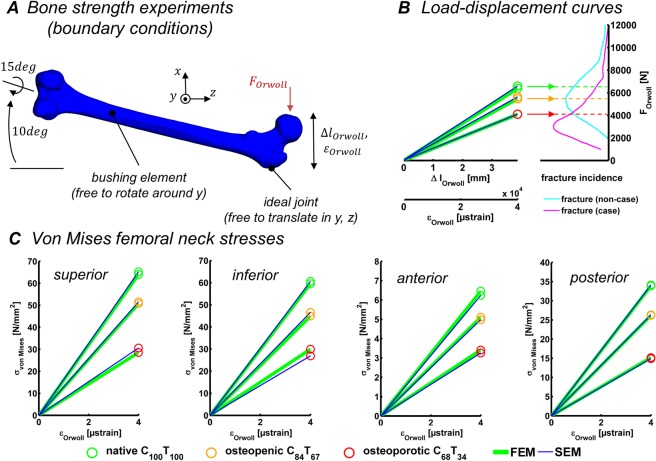
Evaluation of bone quality parameters. (**A**) The boundary conditions of the computational *bone strength* experiments for the fracture risk assessment as defined according to Orwoll, *et al*.^4^ for a sideways fall on the hip. (**B**) The load-displacement curves up to failure (ε_Orwoll_ = 4%) are depicted for the native bone (green marker) and for the weakened bones (orange and red markers). Both, the FEA predictions (green curve) and SEM predictions (blue curve) are shown for validation purposes. For reference, the relative incidence of bone fractures (cyan for non-fracture cases, magenta for fracture cases) is depicted in dependence on the absolute *bone strength* as measured by Orwoll and coworkers. (**C**) The load-displacement curves are generalized to the material characterizing stress-strain curves as depicted for the superior, inferior, anterior, and posterior site of the femoral neck.

To obtain a measure of the hip fracture risk, computational *bone strength* experiments were conducted following the protocol by Orwoll *et al*.^4^. Orwoll and coworkers calculated the *bone strength* (the load at which the fracture occurred) as the applied load from the resulting load-deformation curve at 4% deformation (=4000 µstrain) of the femoral head with respect to the greater trochanter (Fig. 4A,B). This technique was reported to provide excellent predictions of femoral *bone strength* in cadaver laboratory studies (*n = *51, *r*^2^ = 0.80) and an excellent correlation between femoral *bone strength* and osteoporosis-related hip fractures.

Moreover, we modeled osteoporotic bone as described by Little and coworkers^46^, which provided insight into the change of osteogenic parameters during dynamic squat motion for the fracture risk assessment after BMD-loss leading to osteoporotic conditions in the previously analyzed femur specimen. The osteoporotic bone model was parametrically derived from the above described FEM by reducing the Young’s moduli of the cortical and trabecular bone elements to 84% and 67% (referred to as the osteopenic C_84_ T_67_) or to 68% and 34% (referred to as the osteoporotic C_68_ T_34_) in the cortical and trabecular bone compared to the originally QCT-based Young’s moduli assignment, respectively. To verify the osteoporotic bone models, we conducted numerical *bone strength* analyses according to the protocol by Orwoll *et al*.^4^. The load-displacement curves were then generalized to stress-strain curves to allow for fracture risk assessment under more complex and varying boundary conditions (Fig. 4C).

The results of the numerical bone strength experiments exhibit a very good agreement in the load-displacement curves as predicted by the FEA and SEM up to the bone failure criterion of ε_Orwoll_ = 4%. Moreover, the healthy, native bone model (F_Orwoll-FEA_ = 6390 N, F_Orwoll-SEM_ = 6612 N) as well as the parametrically modeled BMD loss-affected bone models C_84_ T_67_ (F_Orwoll-FEA_ = 5480 N, F_Orwoll-SEM_ = 5646 N) and C_68_ T_34_ (F_Orwoll-FEA_ = 4095 N, F_Orwoll-SEM_ = 4110 N) agree with the classification criterions, according to Orwoll *et al*.^4^, which reported the *bone strength* to be an average of µ = 5939 N ± 1919 N for the non-fracture cases and µ = 3782 N ± 1563 N for the fracture cases. As expected, the weakened bone exhibits lower bone strength, which results in higher strains at lower stresses.

### Dynamic stresses and strains during two-leg squat

**Figure 5:**
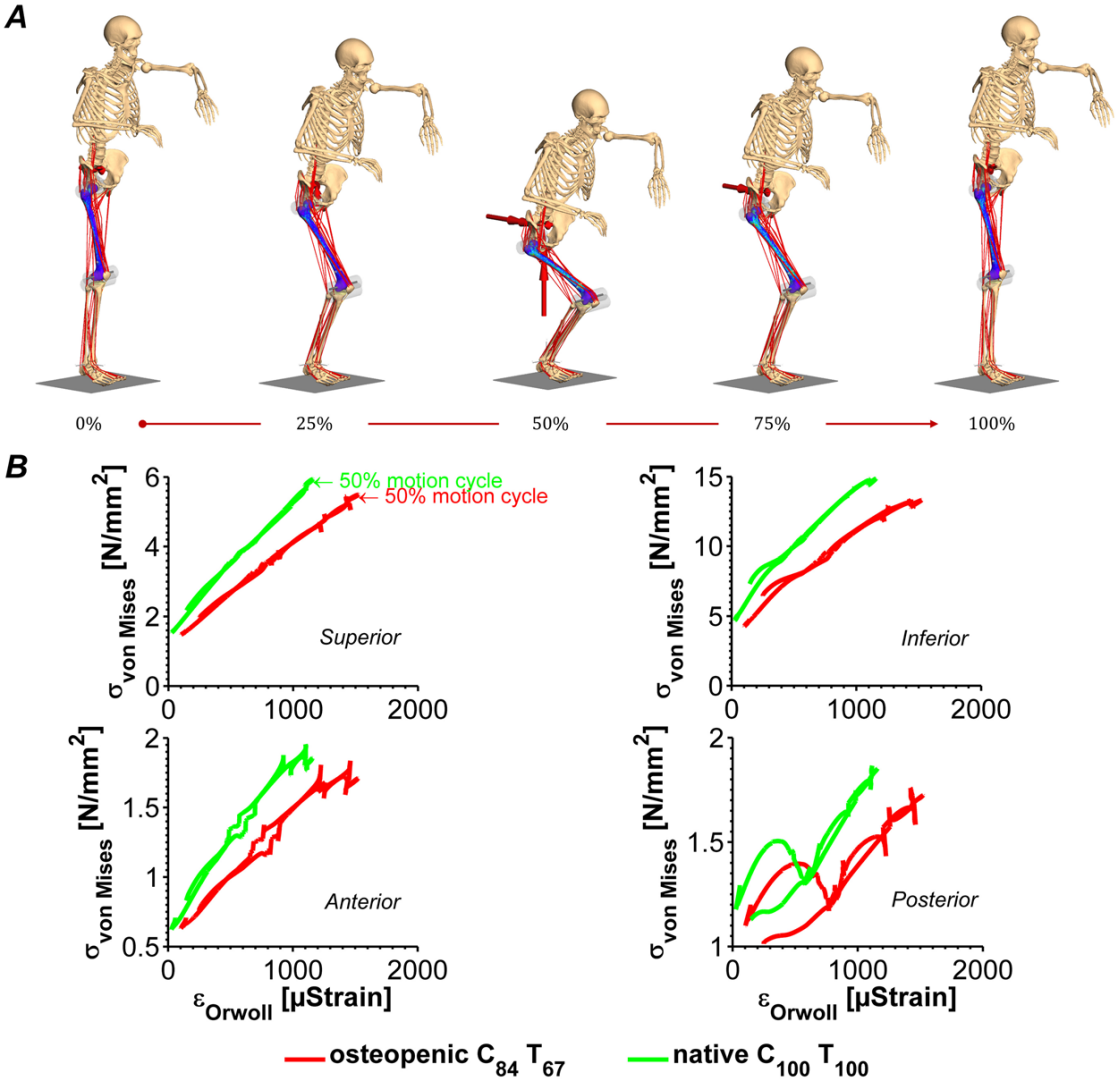
Results of neuro-musculoskeletal flexible multibody simulation of a two-leg squat. The simulation of the entire motion cycle of 4.5 s in real time took 38 s in CPU time (**A**). *Von Mises* stresses are depicted as a function of femoral neck strains (as defined by Orwoll, *et al*.^4^) to allow for *femur strength* characterization (**B**).

The SEMs of the femur were then implemented into a musculoskeletal multibody model (Fig. 5A). For the validation of the musculoskeletal multibody model, please refer to the *Supplementary Information on the validation of the musculoskeletal multibody model* (Fig. SI2). The obtained flexible musculoskeletal multibody model was used to simulate a realistic dynamic two-leg squat, for which the *von Mises* stresses and the strains as defined by Orwoll *et al*.^4^ were calculated as an indicator of hip fracture risk (Fig. 5B).

For hip fracture risk assessment, we implemented the baseline (native C_100_ T_100_) and osteopenic (C_84_ T_67_) SEMs into our NfMBS and compared the in stress-strain curves defined in Fig. 4C as the measure of fracture risk. This enabled us to gain insight into the effect of BMD-loss on the fracture risk measures while including not only specimen-specific bone properties but also NMS dynamics.

The simulation of the entire squat motion cycle of 4.5 s in real time required 38 s of CPU-time. Since the simulated squat motion is an activity of daily living, the bone strains remained in a safe zone, i.e., the strains never exceed the 4% fracture threshold throughout the whole motion cycle, as expected. The osteopenic bone (red curve in Fig. 5), in which the stiffness is reduced to 84% in the cortical and 67% in the trabecular bone exhibits a higher strain and therefore, lower bone strength as compared to the native bone (green curve in Fig. 5). The strain measure that is associated with bone fracture, i.e. the strains in the femoral neck as defined by Orwoll *et al*.^4^, increased by 31.4% owing to osteoporosis-related BMD loss and hence, yielded an elevated risk of femoral hip fractures.

## Discussion

Early, accurate, and efficient bone density assessment is mandatory to tailor and monitor the clinical treatment of, e.g., osteoporosis^5,6,47^. It has been shown that patient-specific FEA that utilizes QCT for material property identification is beneficial over DXA-based BMD measurement for diagnosis and medication effect assessment in osteoporotic patients^2,3,29,30^. However, established techniques, including QCT-based FEA, lack the capability to estimate stresses and strains that arise due to the dynamic multiaxial loading of the bone under varying loading conditions as usually present in daily living activities and specific to the individual NMS system.

To address these limitations, we introduced a novel, clinically-motivated workflow to enable neuro-musculoskeletal flexible multibody simulation (NfMBS) that features dynamic stress and strain computation (Fig. 1) by incorporating modally reduced flexible SEMs into conventional, subject-specific NMBS to enhance clinicians’ diagnostic repertoire on BMD loss-related bone fractures. We present a validated workflow that establishes a general method which can estimate dynamic femoral bone stresses and strains, non-invasively. This method can help in developing bone mechanoregulation theories and in identifying various risk scenarios of femoral bone failure and specifically, femoral hip fractures.

The major contributions of our work are the integration of a subject-specific, QCT-based FEA of the human femur into the workflow of patient-specific NMBS together with its validation. The inclusion of structural patient specificity, i.e. morphological and material properties of the bone, and functional patient specificity, i.e. musculoskeletal architecture and motion dynamics of the NMS system, is necessary to enable clinically relevant NfMBS to identify early tendencies towards femoral hip fractures as it is a major risk scenario. We could show that our QCT-based FEM translated very well to the flexible SEM for its implementation into NfMBS, while maintaining the capability to reproduce osteoporosis-related bone weakening under dynamic NMS boundary conditions. The comparison of FEM and SEM exhibited negligible deviations between the target parameters at the femoral neck in terms of *von Mises stresses* and *bone strength*. In this context, our experimentally validated *in-silico* analysis of the human femur under loading conditions normally seen in daily living activities is the first study of its kind and provides great insight into highly prevalent femoral hip fractures, which are practically impossible to analyze *in-vivo*. Although the bone quality was varied only parametrically from healthy (C_100_ T_100_) to osteopenic (C_84_ T_67_) to osteoporotic bones (C_68_ T_34_), the results seem representative of osteoporosis-related fractures, since the predicted femoral *bone strengths* were in very good agreement with the literature data^4,5,48^. Our results provide confidence that NfMBS could predict the individual *bone strength* (as measured in Orwoll *et al*.^4^) as a major diagnostic parameter for the assessment of risks and medication effects under physiological-like conditions in an accurate and efficient manner. Notably, is has been shown that there can be more complex criteria derived from the predicted mechanical stresses and strains for quantifying the resistance of the femoral bone against failure^49–51^.

Our most important limitation was that our study has a rather methodological character with its emphasis on the validity of the proposed workflow. A limitation of the presented work is that only one healthy femur specimen was analyzed. From the clinical point of view, it would be, therefore, desirable to apply the workflow to a larger, statistically relevant number of osteoporotic bone specimens and eventually to patient cohorts.

However, an extensive body of studies has shown that QCT-based FEA is a powerful diagnosis tool that possesses superior accuracy and reliability in the assessment of *bone strength* and bone fracture localization over DXA-based BMD measurements^5,6,29,30,47^. As mentioned above, BMD alone is not sufficient to describe the *in-vivo* intricacies that involve bone morphology and material-structural bone properties^47^. Thus, the logic extension to QCT-based FEA is the integration of patient-specific NMS dynamics. Clearly, the risk of bone fractures could be more accurately diagnosed with the complementation of NfMBS-predicted bone strength data under physiological-like representation with skeletal dynamics and soft tissue interaction^7,8^. NfMBS provides much deeper insight into relevant risk factors, as NMS dynamics—such as soft tissue damage or motor control dysfunctions—can be included into the set of boundary conditions to ultimately better resemble the patient-specific *in-vivo* situs. Consequently, NfMBS could enhance key aspects of QCT-FEA to ultimately arrive at a predictive tool that enables a combined *in-silico* analysis protocol of BMD measurement and dynamic NMS computer simulation for an improved diagnosis, e.g., of osteoporosis.

In fact, the direct comparison of our experimentally validated QCT-based FEM against the flexible SEM, in terms of the prediction accuracy and efficiency of clinically relevant bone parameters, revealed that NfMBS can handle the trade-off between these aspects extremely well, i.e., the SEM preserved very high accuracy while outperforming the dynamic QCT-based FEA in terms of magnitude in CPU-time. It should be noted that, currently, only NfMBS possesses the capability to sample the enormous parameters space for clinically relevant solutions by running numerous non-invasive parameter analyses on the patient’s NMS system under dynamic boundary conditions^32^. Contrarily, very complex load cases, like the forward dynamic NMS squat simulation, have not been achieved up till date, by the use of FEA.

Regarding the QCT-based FEA, our FEM resembled the structure-mechanical properties of the tested bone specimen by, first, reconstructing the bone specimen’s morphology using high-resolution QCT and then, discretizing the 3D solid into quadratic tetrahedral finite elements that were assigned bone-specific BMD-dependent Young’s moduli for inhomogeneous and HU-value dependent, but isotropic and linear-elastic, material properties. The structural behavior of the bone in the plastic strain range beyond the yield point, especially the bone’s failure mechanism itself, has not been investigated within this study and is not sufficiently described using a linear-elastic material model. However, the *bone strength* assessment according to Orwoll *et al*.^4^ is applicable: the stress-strain curve of the femoral bone is roughly linear until the ultimate load is reached^17,18,48,52,53^, which should never occur during daily living activities. Generally, the consideration of microstructural processes along a timely trajectory, e.g. the architectural change of the trabecular bone, cannot be easily predicted using NfMBS. After capturing the patients current NMS condition, the clinically-relevant predictive capacity of NfMBS lies more in the incorporation of structure-mechanical and NMS boundary conditions for rapid and extensive osteogenic parameter analysis than in the inclusion of overly complex FEMs, as simple macroscopic QCT-based FEAs have been proven efficient^17,18,29,52,53^. Moreover, it was shown that there is only a marginal difference in the mechanical response when using anisotropic material models^54^.

However, our results show that decreases in BMD, e.g. due to osteoporosis or lytic bone changes, lead to abnormalities in the stresses and strains, which indicates a higher risk of fractures especially around the femoral neck. This agrees with numerous studies on the fracture risk of osteoporotic bone and its clinical prevalence, which report femoral hip fractures as the most prevalent osteoporosis-related fracture^4,9,48^. In this context, NfMBS resolves the trade-off between the predictive capability of fracture risk/fracture site and computational efficiency.

As such, our approach might be particularly valuable in the regular assessment of patient-specific *bone strength*, i.e., as an early diagnosis tool of fracture risk before the possibility of a fracture arises. In this respect, Anitha *et al*.^47^ has shown that even low-quality CT imaging does not affect the prediction capability of QCT-based FEA. Hence, more frequent follow-up CT scans in combination with NfMBS would allow more regular monitoring and earlier treatment of BMD loss-related bone conditions.

Further limitations apply to the incorporation of flexible SEMs of bone structures into NMBS and the NMBS itself as follows: idealizations and simplifications of the real biomechanical system are inherent to NMBS that uses the multibody formalism to approximate the system’s dynamics by means of rigid bodies, geometric constraints and discrete force elements. An overview can be found in^31,32^. We took great care of the representation of the musculoskeletal geometry, i.e. bone morphology, kinematic topology, and muscle attachment sites, throughout the entire modeling process, since musculoskeletal architecture is known to have considerable impact on muscle-driven motion patterns and joint loading^55,56^, and most likely on bone loading as well. For validation purposes, the entire workflow was specifically based on experimental datasets^45,55^, and the musculoskeletal architecture, in particular, was refined in close collaboration with an orthopaedic surgeon. Additionally, we validated the boundary conditions due to musculoskeletal dynamics in terms of the quadriceps force^57,58^ and the resultant hip reaction force^59^ (Fig. SI2).

As it concerns the neuro-muscular control, the computed muscle control algorithm may allow simulation of muscle-induced motion; however, the solution of using static optimization techniques for muscle distribution problems, although common in musculoskeletal multibody dynamics, is still a matter of debate in terms of neurophysiological evidence. In order to obtain dynamic stress and strain distributions for patient-specific and load case dependent scenarios, it is not only necessary to include bone-specific material properties but also subject-specific musculoskeletal architecture, i.e., muscle forces that act on the skeletal system as they would *in-vivo*^8^. Accomplishing this is still challenging as direct muscle force measurement is not possible and although statistical methods exist, automated implementation of musculoskeletal architecture is an unresolved problem^40^. Passive forces due to muscular or capsular structures were not considered, since passive soft tissue accounts for less than 10% of intersegmental torques during comparable activities^60^.

The use of publicly available datasets (SimTK^45^, TLEM 2.0^55^, Bergmann *et al*.^59^) and the complementation of these data by our own experiments allowed for efficient validation of our workflow. This, however, stresses the importance of research on methods that help to identify an individuals’ musculoskeletal system on the way toward clinically-relevant NfMBS^40^. Despite limited technical or methodological challenges, a considerable effort must be directed toward the clinical integration of QCT-based FE modeling and, ultimately, of NfMBS.

By defining a symmetry condition between the pelvis and the anatomical sagittal plane, we simplified the motion of the contralateral side. Kinematic studies suggest that this simplification is valid as humans show equivalent kinematics in the contralateral side during sitting down and standing up activities^61^. The two-leg squat, in particular, is a rather symmetrical motion and the symmetry condition in the sagittal plane showed only minor reaction forces in the constraint directions. This suggests that there was no abnormal bending of the femur.

In summary, we introduced a novel workflow for subject-specific NfMBS featuring dynamic stress and strain computation. The workflow aims at the efficient integration of the capabilities of QCT-based FEA into established subject-specific NMBS. Our work provides the foundation for a clinically motivated workflow that incorporates QCT-based FEMs into subject-specific NMBS. The performance of the novel workflow is supported by experimental results on a fresh human femur specimen and is in good agreement with comparable studies. Our results provide confidence that NfMBS can provide a computational tool of predictive *in-silico* analysis that could help in developing bone mechanoregulation theories, in identifying various risk scenarios and ultimately in optimal treatment options for femoral bone failure; especially in terms of highly problematic femoral hip fractures due to BMD loss. Until clinical translation, however, numerous challenges that are mainly associated with musculoskeletal modeling, e.g. an automatized implementation of subject-specific musculoskeletal architecture, need to be overcome. The application of NfMBS to a larger number of specimens, including osteoporotic bone specimens, is currently in progress. NfMBS seems promising in providing a generalized and reliable framework for the assessment of fracture risk and treatment options in osteoporotic patients.

## Materials and Methods

For our NfMBS that features dynamic stress and strain computation, we used the TCP/IP communication framework that comprised the server SIMPACK® (v9.7, Dassault Systèmes Deutschland GmbH, Gilching, Germany), in which the NMS simulation model with a flexible femur SEM was implemented, and the client Matlab/Simulink® (v8.1, 2013a, The MathWorks Inc., Natick, USA), in which the computed muscle control was implemented. All simulations were run on an off-the-shelf desktop PC (Intel® Core™ i7-4790 CPU @3.60 GHz 8.00 GB RAM).

### Experimental testing of a fresh human femur

**Figure 6:**
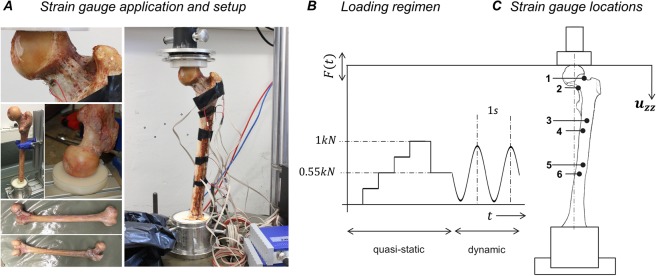
Experimental testing of a fresh human femur specimen. For validation purposes of the above workflow, static and dynamic axial compression tests were conducted on the thawed fresh femur (**A**). The displacement of the force application point and the linear strains within the bone’s elastic deformation range were measured under static and dynamic loading regimen (**B**). The linear strains were applied to the cortical bone at six locations on the femoral neck and proximal shaft (**C**).

We conducted static and dynamic axial compression tests on a thawed fresh femur with the experimental protocol adapted from^15^ (Fig. 6). The specimen was received through an anatomic gift program (Science Care Inc., Phoenix, Arizona, USA) and was authorized by the ethical review committee (Ethics Committee of the Bavarian Medical Association, BLAEK 2011-058, Munich, Germany). We confirm that all experiments and research were performed in accordance with relevant guidelines and regulations, and informed consent for study participation was obtained from the donor. The experimental protocols for measurements on the human femur specimen were approved by the cluster ‘Numerical Simulation’ within the Musculoskeletal Biomechanics Network (MSB-Net) of the Basic Research section of the German Society for Orthopaedics and Trauma (DGOU).

We measured the global deformation, i.e. the displacement of the force application point, and the local strains, i.e. the strain distribution in the cortical bone, in response to the compression within the bone’s elastic deformation range by using linear strain gauges. The detailed experimental protocol can be found in *Supplementary Experimental Protocol of the in-vitro analysis*. Note that mechanical testing is not mandatory to the proposed workflow and served validation purposes only.

### Quantitative CT scan of a fresh human femur specimen

As depicted in Fig. 6, the femur, with its entire soft tissue structures removed, underwent a quantitative CT (QCT) scan for image-based FEM and SEM generation. The CT scan was conducted at the Trauma Center Murnau (SOMATOM Definition AS + CT scanner, Siemens AG, Erlangen, Germany) and included a BMD calibration phantom (European Forearm Phantom-05-83, QRM GmbH, Möhrendorf, Germany) for image-based material property assignment and calibration purposes. Specifically, the phantom served as quantification of the distributed bone stiffness via relating material specific X-ray attenuation in Hounsfield units (HU) to BMD and ultimately to the distributed Youngs’ moduli.

The reconstruction of the CT scans to the 3D FEM followed the protocol described in^14^. The CT images were saved as DICOM files with a resolution of 0.29 mm × 0.29 mm and a slice thickness of 0.6 mm, resulting in 886 layers. The DICOM files were imported in the segmentation software package AMIRA^®^ (v5.4.1, FEI Visualization Sciences Group, Hillsboro, Oregon, USA) to reconstruct the 3D bone surface model using triangulated surfaces. The resulting surface model was smoothed and converted into a surface of analytical non-uniform rational B-splines using Geomagic Studio (v2013, 3D Systems, South Carolina, U.S.A.), and finally exported to the FE pre-processor via the IGES interface.

### Finite-element modeling

In this pre-processing step, the optimized 3D surface model was converted into a 3D solid model and meshed with quadratic, 10-node, tetrahedral finite elements (C3D10) using Abaqus/CAE® (v6.13-1, Dassault Systèmes Simulia Corp., Providence, Rhode Island, USA).

As we were considering clinically relevant load cases with rather small deformations, we chose a linear-elastic HU-dependent material model. More specifically, our in-house mapping algorithm assigns a Youngs’ modulus *E*_i_ to each node i of the FEM that is in dependence on the HU values obtained from the QCT scans of the very same femur at the respective node location. To do so, the calibration phantom with a known BMD served as a reference for the linear regression relating the HU value of the phantom to the known BMD’s equivalent ${{\rho }}_{{\rm{ash}}}$, which is the so-called ash density:

1 $${{\rho }}_{{\rm{ash}}}={HU}/\mathrm{895.93.}$$

The bone’s Young’s modulus could then be assigned nodewise, according to its respective ash density, following another relation presented in Cong *et al*.^62^:

2 $${{E}}_{{\mathbb{i}}}=20000\,{{\rm{e}}}^{{\rm{a}}{{\rm{e}}}^{{{\rm{b}}}_{{\rm{i}}}}}\,{\rm{with}}\,{\rm{a}}=-\,5.19\,{\rm{and}}\,{{\rm{b}}}_{{\rm{i}}}=-\,2.30{{\rho }}_{{\rm{ash}},{\rm{i}}},$$

where *E*_i_ is the Young’s modulus in GPa and ${{\rho }}_{\mathrm{ash},{\rm{i}}}$ is the ash density in g/cm^3^ for the respective FE-node ${\rm{i}}$. A Poisson’s ratio of ${\nu }=0.3$ was assumed over the entire bone^46^. Note that values below 1 MPa were set to 1 MPa, to avoid negative or zero elasticities^63^.

The mesh quality was optimized within a convergence analysis: the element size was incrementally decreased from 7.4 mm to 2.0 mm until the change in the computed strains and deformations fell below 5%. The final mesh for our baseline model consisted of 123,454 nodes and of 83,845 elements with an element size of 3.8 mm.

The numerical boundary conditions and locations of the strain gauges were extracted from the 3D scan to ensure that they resembled the experimental setup. To go into further detail: The FE nodes of the distal part of the femoral cortex below the casting resin surface were entirely constrained to zero DoF, and a compression load similar to the experimental loading was applied to a reference point that kinematically distributed the resulting displacement to the FE nodes on the upper hemisphere of the femoral head.

Based on the described static FE model, the femur specimen was also analyzed within a dynamic FEA with an implicit solver over 10 s simulation time. A dynamic loading regimen was defined and a constant mass density of 1940 kg/m³ was assumed^64^. The dynamic loading regimen was implemented as sinusoidal function equivalent to the experimental loading regimen as described in *Supplementary Information on the experimental protocol of the in-vitro analysis*. The resulting force was applied at the femoral head to a reference point that was directly coupled to the FE nodes of the femoral head via kinematic coupling representing the experimental stamp.

### Superelement model

Since clinically relevant load cases only cause small linear-elastic deformations in the bone that otherwise undergo large rigid body motion, the *Craig-Bampton modal reduction method*^42^ in the *floating frame of reference formulation* was employed for the SEM generation from the specimen-specific FEM.

The unreduced FEM,

3 $${\boldsymbol{M}}\,\ddot{{\boldsymbol{u}}}+{\boldsymbol{K}}\,{\boldsymbol{u}}={\boldsymbol{f}}$$

is converted into the SEM by projecting the large number of FE-related DoFs into reduced matrices of mass $\tilde{{\boldsymbol{M}}}$, stiffness $\tilde{{\boldsymbol{K}}}$ and modal coordinates $\tilde{{\boldsymbol{u}}}$ using *Craig-Bampton* modes, ${{\boldsymbol{\Phi }}}_{{\rm{CB}}}$ with ${\boldsymbol{u}}={{\boldsymbol{\Phi }}}_{{\rm{C}}{\rm{B}}}\mathop{{\boldsymbol{u}}}\limits^{ \sim }$:

4 $$\mathop{\underbrace{{{\boldsymbol{\Phi }}}_{{\rm{C}}{\rm{B}}}^{{\rm{T}}}{\boldsymbol{M}}{{\boldsymbol{\Phi }}}_{{\rm{C}}{\rm{B}}}}}\limits_{\mathop{{\boldsymbol{M}}}\limits^{ \sim }}\ddot{\mathop{{\boldsymbol{u}}}\limits^{ \sim }}+\mathop{\underbrace{{{\boldsymbol{\Phi }}}_{{\rm{C}}{\rm{B}}}^{{\rm{T}}}{\boldsymbol{K}}{{\boldsymbol{\Phi }}}_{{\rm{C}}{\rm{B}}}}}\limits_{\mathop{{\boldsymbol{K}}}\limits^{ \sim }}\mathop{{\boldsymbol{u}}}\limits^{ \sim }=\mathop{\underbrace{{{\boldsymbol{\Phi }}}_{{\rm{C}}{\rm{B}}}^{{\rm{T}}}{\boldsymbol{f}}}}\limits_{\mathop{{\boldsymbol{f}}}\limits^{ \sim }}.$$

The mode shapes ${{\boldsymbol{\Phi }}}_{{\rm{CB}}}$ account for static and dynamic deformations due to forces, constraints, and joints acting upon the SEM, but this is, however, only within a limited frequency range. As a result, the mode shapes ${{\boldsymbol{\Phi }}}_{{\rm{C}}{\rm{B}},k}$ that correspond to higher frequency responses of the SEM can be truncated without a relevant loss of information. The reduced SEM, i.e., the matrices $\tilde{{\boldsymbol{M}}}{\boldsymbol{,}}\,\tilde{{\boldsymbol{K}}}$, the modal coordinates $\tilde{{\boldsymbol{u}}}$, and the modes shapes ${{\boldsymbol{\Phi }}}_{{\rm{CB}}}$, were calculated within a *Craig-Bampton* mode shape analysis using Abaqus/CAE®.

**Figure 7:**
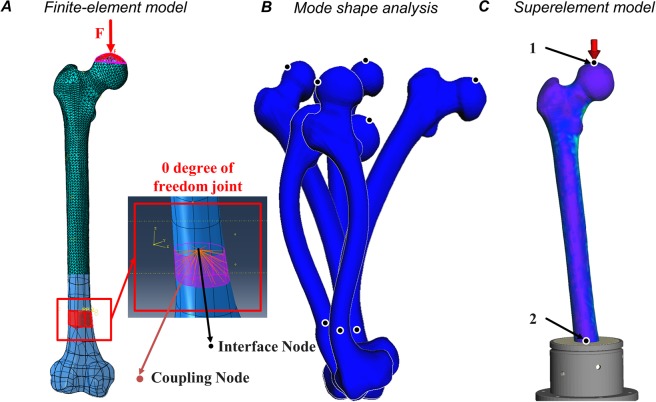
Domain-specific models of the human femur for the experimental setup according to Fig. 6. The finite-element model (**A**) is modally reduced using shape functions obtained from a *Craig-Bampton*^42^ mode shape analysis (**B**) to a flexible superelement model (**C**). In the finite-element model, the clamping is modeled by a zero degree of freedom joint. Implemented into the multibody simulation framework, the superelement model interfaces the multibody system via interface nodes on which forces and torques are exerted upon. In the model of the experimental setup shown in (**C**), the interface nodes are the load force application point 1 and the fixed clamping 2. In the musculoskeletal model shown in Fig. 8, interface nodes are provided for each joint marker, each muscle attachment site, and each via-point, respectively.

Finally, the flexible bone structure was integrated via the *flexible body input* interface into the multibody dynamics software environment SIMPACK® for subsequent, flexible multibody dynamic analysis. The numerical boundary conditions were defined similarly to the FEM (Fig. 7). All interface nodes (i.e. the respective reference point in the FE model) were connected to the respective subset of coupling nodes (i.e. the respective nodes of the FE model for the definition of the respective boundary conditions) using structural-continuum distributed coupling, which allows the coupling nodes to move relative to each other and reduces the impact of boundary conditions on the calculation of stress and strain.

As required for dynamic multibody analysis, a constant mass density of 1940 kg/m^3^ equivalent to the dynamic FEA was assumed^64^, the number of mode shapes was set to 30, and a proportional damping ratio of 0.025 was selected for all modes. Note that the number of mode shapes and the proportional damping ratio were verified by means of the dynamic compression tests and *bone strength* experiments.

As it concerns the stress and strain computation, the SEM’s stresses are recovered by deploying a linear-elastic material law. According to^65^, the (6, 1) vector of stresses ${\boldsymbol{\sigma }}$ in a point of the flexible body is related to the (6, 1) vector of strains ${\boldsymbol{\varepsilon }}$ by ${\boldsymbol{\sigma }}={\boldsymbol{E}}\,{\boldsymbol{\varepsilon }}$ with the symmetric (6, 6) matrix of elastic coefficients ${\boldsymbol{E}}$. With the strain vector ${\boldsymbol{\varepsilon }}$ defined by the partial derivatives of the deformation vector ${\boldsymbol{u}}$ according to ${\boldsymbol{\varepsilon }}={\boldsymbol{Du}}$ using the *(6, 3)-* partial derivative operator matrix ***D*** and ${\boldsymbol{u}}={{\boldsymbol{\Phi }}}_{{\rm{CB}}}\tilde{{\boldsymbol{u}}}$ with the Craig-Bampton shape functions ${{\boldsymbol{\Phi }}}_{{\rm{CB}}}$ used in (4), the stress vector ${\boldsymbol{\sigma }}$ is related to the modal coordinates $\tilde{{\boldsymbol{u}}}$ by

5 $${\boldsymbol{\sigma }}={\boldsymbol{E}}\,{\boldsymbol{D}}\,{{\boldsymbol{\Phi }}}_{{\rm{CB}}}\,\tilde{{\boldsymbol{u}}}{\boldsymbol{.}}$$

### Musculoskeletal multibody model

**Figure 8:**
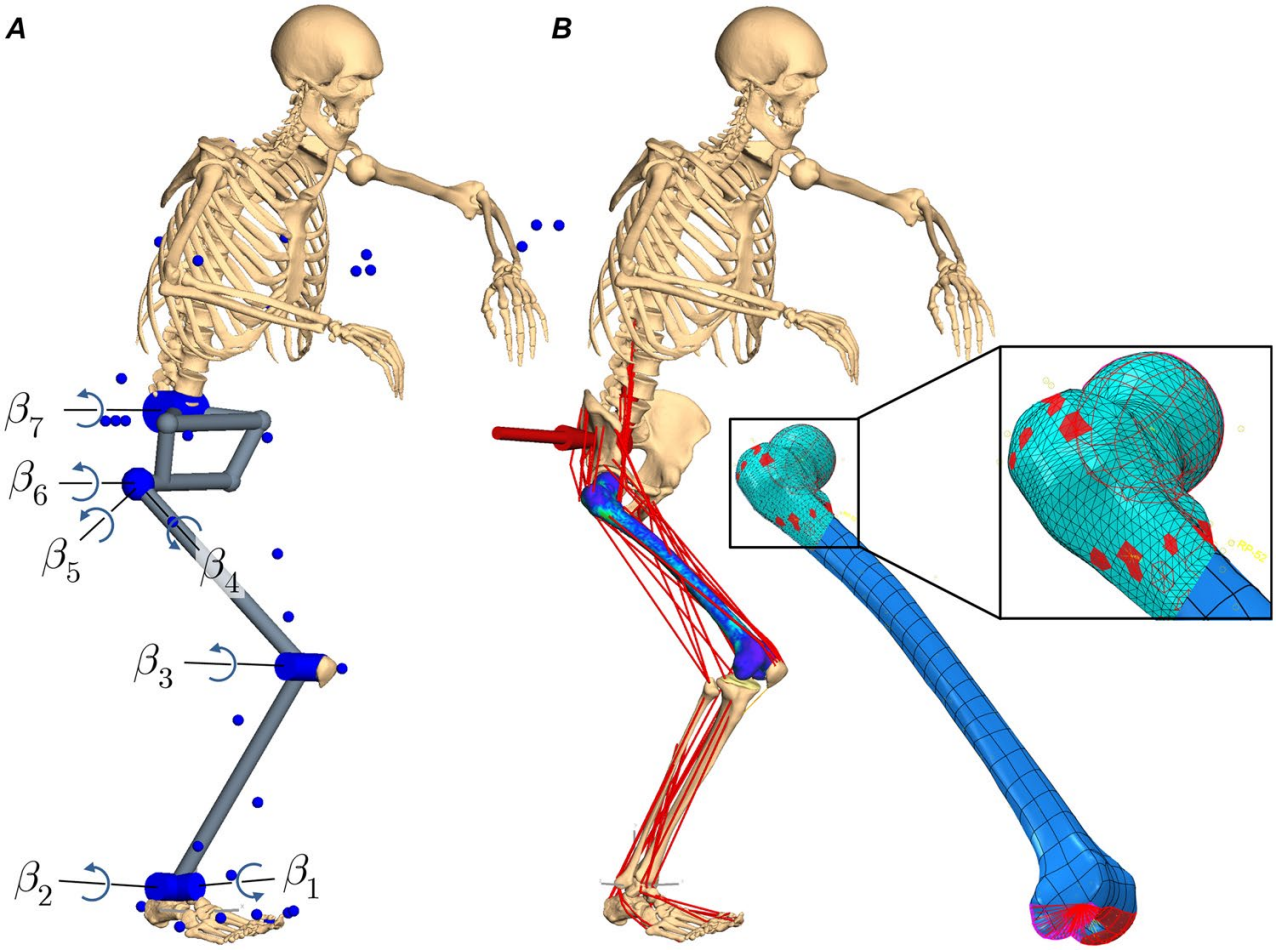
Musculoskeletal multibody model featuring the flexible femur superelement. (**A**) The skeletal multibody model that was used for inverse kinematic analysis to convert Cartesian marker trajectories into joint space ${\boldsymbol{\beta }}$ for the equation of motion. (**B**) The musculoskeletal multibody model as described in detail in continuous text. Since the dynamic motion is muscle driven, spring-damper force elements restricted the motion of the pelvis to the sagittal plane and thus, resembled the reaction forces of the lower-left extremity for the two-leg squat. The femur superelement was incorporated using a master node for each joint marker, each muscle attachment site and each via-point, respectively.

The NfMBS model (Fig. 8) was based on two complementary experimental datasets. The first dataset consisted of the experimentally validated SEM of the human femur specimen, as described above. The second dataset was obtained from the SimTK website (https://simtk.org/home/kneeloads) as made available to the public within the 4^*th*^*Grand Challenge Competition to Predict In Vivo Knee Loads*^45^. To put it briefly, the *SimTK* dataset is a comprehensive and consistent collection of subject-specific models and measurements from one male subject (age = 88 yrs, height = 168 cm, m = 66.7 kg) that enables the validation of NMBS estimates in the lower extremity. The database included the geometry of the lower-right extremity (pelvis, femur, patella, tibia, fibula, and pes) from CT scans (pre- and post-op) and marker trajectories from motion capture experiments. To implement the femur specimen SEM into the NMBS, bone geometries and marker trajectories of the SimTK dataset were globally scaled to the size of the femur specimen (111.53%) using Geomagic Studio.

The reconstructed bone segments were mutually linked by ideal joints to an open kinematic chain (Fig. 8A). Starting from the ground, the inertia-fixed pes was linked via a two DoF universal joint to the tibia and fibula, which were summarized as one single segment. Similarly, the tibia-fibula compound was connected via one DoF hinge joint to the flexible femur SEM, which was in turn connected to the pelvis via a three DoF spherical joint. Finally, the upper body^66^ was linked via a one DoF revolute joint to the pelvis’ sacrum endplate center. In addition, spring-damper force elements (referred to as *symmetry condition*) restricted the motion of the sagittal plane by exerting forces and torques to the pelvis with respect to the ground, thus, eliminating abnormal bending torques around the sagittal plane during a symmetric squat motion. In this manner, the lower-left extremity was represented by its reaction forces and torques, acting upon the pelvis.

The preservation of the bone geometry and anatomical landmarks allowed for the definition of mass properties, for the identification of joint rotation axes, by fitting spheres or cylinders into the articulating surfaces of adjacent segments, and moreover, for the establishment of standardized reference systems^67,68^, which allowed for the description of joint dynamics and the identification of the attachment sites of relevant muscle structures of the lower extremity as documented in the *Twente Lower Extremity Model 2.0* dataset^55^. The origins and insertions of the muscles, in particular, were verified by an experienced orthopaedic surgeon. Since a single bone segment was defined as the sum of its corresponding soft tissue and bone structures, soft tissue masses could be estimated, based on the subject’s mass using regression techniques^69^.

Relevant muscles were implemented in the form of unidimensional Hill-type force elements acting along their anatomical attachment sites. To account for the exertion of physiological forces, the functional units of muscles were subdivided into several force elements to account for wide attachment areas^55^. Deflection phenomena from muscles around the bone were considered by deploying segment-fixed via-points and wrapping^70^. A complete list of muscle structures with their Hill-type model parameters is summarized in the Supplementary Table SI1. Passive forces due to muscular or capsular tissue have been neglected within this work, since they contribute less than 10% to intersegmental torques during comparable activities^60^.

Joint markers and muscle attachment sites—including muscle via-points—adjacent to the flexible femur SEM, required the definition of interface nodes in the FE modeling step as they potentially deform the femoral bone and thus, must be considered during modal reduction. As it concerns the joint markers, the subsets of FE-nodes were defined according to the contacting cartilage surfaces of the femoral head and condyles. As it relates to the muscle attachment sites, the FE-nodes located within a radius of approx. 0.5 cm around the respective attachment site were defined as the interface node’s subset of respective coupling nodes. The choice of the radius of 0.5 cm results from FE model’s element size of 3.8 mm and the fact that the attachment site area is accounted for by the subdivision of the whole muscle into functional subunits as defined by Carbone *et al*.^55^ and not the area of each subunit itself.

### Inverse kinematics

The two-leg squat motion scenario as available from the *SimTK* dataset^45^ comprises the trajectories of reflective skin markers that were applied to the male subject’s body at prominent anatomical landmarks to track its motion. The subject performed three squat cycles with a self-selected speed. Utilizing the kinematic topology of the NMS model as defined above, the marker motions and the resulting overall motion of the rigid skeletal multibody system were converted into trajectories of the minimal coordinates in joint space ${\boldsymbol{\beta }},\dot{{\boldsymbol{\beta }}},\mathop{{\boldsymbol{\beta }}}\limits^{..}$ resulting in a total of seven relative joint coordinates of the kinematic chain. The experimentally obtained joint trajectories were then used as the input for the inverse dynamics and computed muscle control to generate coordinated muscle forces and in turn, the desired squat motion.

### Inverse dynamics and computed muscle control

In our NfMBS featuring dynamic stress and strain computation, we deployed a computed muscle control (CMC) algorithm^71^ to track the experimentally obtained joint trajectories ${({\boldsymbol{\beta }},\dot{{\boldsymbol{\beta }}},\mathop{{\boldsymbol{\beta }}}\limits^{..})}_{\exp }$ by means of coordinated muscle forces. For CMC, our inverse dynamics model,

6 $${{\boldsymbol{\tau }}}_{{\rm{m}}}({\boldsymbol{\beta }},\dot{{\boldsymbol{\beta }}},\ddot{{\boldsymbol{\beta }}},{\boldsymbol{a}})={\boldsymbol{M}}({\boldsymbol{r}},{\boldsymbol{\beta }})\,{\ddot{{\boldsymbol{\beta }}}}_{\exp }-({{\boldsymbol{\tau }}}_{{\rm{c}}{\rm{c}}}({\boldsymbol{r}},{\boldsymbol{\beta }},\dot{{\boldsymbol{\beta }}},\ddot{{\boldsymbol{\beta }}})+{{\boldsymbol{\tau }}}_{{\rm{g}}}({\boldsymbol{r}},{\boldsymbol{\beta }}))$$

is used for input-output linearization of the NMS system. On the right-hand side of (6), the overall inertial torque which comprises the inertia torque ${\boldsymbol{M}}(\,\cdot \,)\,\ddot{{\boldsymbol{\beta }}}$ appears due to the acceleration ${\ddot{{\boldsymbol{\beta }}}}_{\exp }$ and the torque ${{\boldsymbol{\tau }}}_{{\rm{c}}{\rm{c}}}(\,\cdot \,)$ which are due to Coriolis and centrifugal effects and the torque due to gravity ${{\boldsymbol{\tau }}}_{{\rm{g}}}(\,\cdot \,)$. The vector ${\boldsymbol{r}}$ is a set of absolute coordinates that describe the actual spatial positions of the bodies, relative to a fixed inertial reference system ${{\mathscr{C}}}_{0}$. On the left-hand side of (6) the torques due to active muscle forces ${{\boldsymbol{\tau }}}_{{\rm{m}}}(\,\cdot \,)$ appear.

The NMS system dynamics is then superposed with a generic feedback control,

7 $$\ddot{{\boldsymbol{\beta }}}={\ddot{{\boldsymbol{\beta }}}}_{\exp }+{{\boldsymbol{K}}}_{D}\,{\dot{{\boldsymbol{e}}}}_{{\boldsymbol{\beta }}}+{{\boldsymbol{K}}}_{P}\,{{\boldsymbol{e}}}_{{\boldsymbol{\beta }}}\,{\rm{w}}{\rm{i}}{\rm{t}}{\rm{h}}\,{{\boldsymbol{e}}}_{{\boldsymbol{\beta }}}={\boldsymbol{\beta }}-{{\boldsymbol{\beta }}}_{\exp },\,{\dot{{\boldsymbol{e}}}}_{{\boldsymbol{\beta }}}=\dot{{\boldsymbol{\beta }}}-{\dot{{\boldsymbol{\beta }}}}_{\exp }$$

to accurately track experimentally obtained motion maneuvers ${({\boldsymbol{\beta }},\dot{{\boldsymbol{\beta }}},\mathop{{\boldsymbol{\beta }}}\limits^{{\boldsymbol{..}}})}_{\exp }$. By choosing the controller gains ${{\boldsymbol{K}}}_{D}={\rm{d}}{\rm{i}}{\rm{a}}{\rm{g}}({k}_{D,1},...,{k}_{D,f})$ to ${k}_{D,i}=2\sqrt{{k}_{P,i}}\,$ and ${{\boldsymbol{K}}}_{P}={\rm{d}}{\rm{i}}{\rm{a}}{\rm{g}}({k}_{P,1},...,{k}_{P,f})$ to ${k}_{P,i} > 0$, the tracking errors will converge to zero in a critically-damped manner.

The resulting net torques ${{\boldsymbol{\tau }}}_{{\rm{m}}}$ around the respective DoF is then distributed over the available musculotendon actuators ${{\boldsymbol{f}}}_{{\rm{m}},i}(\,\cdot \,)$ by using static optimization^72^,

8 $$\mathop{min}\limits_{a}Z({\boldsymbol{a}})\equiv {{\boldsymbol{a}}}^{{\rm{T}}}\,{\boldsymbol{V}}\,{\boldsymbol{a}}{\mathbb{,}}\,\,{\rm{s}}{\rm{u}}{\rm{b}}{\rm{j}}{\rm{e}}{\rm{c}}{\rm{t}}\,{\rm{t}}{\rm{o}}\,{\boldsymbol{D}}\,{\boldsymbol{a}}\,=\,{{\boldsymbol{\tau }}}_{{\rm{m}}}\,{\rm{a}}{\rm{n}}{\rm{d}}\,0\le {a}_{i}\le 1,$$

where $\mathop{min}\limits_{{\boldsymbol{a}}}Z({\boldsymbol{a}})$ refers to an energy-optimal quadratic cost function that is solved for the optimal activation levels ${a}_{i}^{\ast }$ and $0\le {a}_{i}\le 1$ that are the physiological bounds to the optimization problem in (8). The diagonal weight matrix ${\boldsymbol{V}}={\rm{d}}{\rm{i}}{\rm{a}}{\rm{g}}({V}_{1},...,{V}_{n})$ contains the muscle volumes ${V}_{{\rm{i}}}$. The hard constraints ${{\boldsymbol{\tau }}}_{{m}}\,=\,{\boldsymbol{D}}{\boldsymbol{a}}$ represent the linear system of equations formulated in (8),

9 $${{\boldsymbol{\tau }}}_{{\rm{m}}}({\boldsymbol{\beta }},\dot{{\boldsymbol{\beta }}},\ddot{{\boldsymbol{\beta }}},{\boldsymbol{a}})=\mathop{\underbrace{[\begin{array}{ccc}{D}_{1,1} & \cdots  & {D}_{1,n}\\ \vdots  & \ddots  & \vdots \\ {D}_{7,1} & \cdots  & {D}_{7,n}\end{array}]}}\limits_{{\bf{D}}}\mathop{\underbrace{[\begin{array}{c}{a}_{1}\\ \vdots \\ {a}_{n}\end{array}]}}\limits_{{\bf{a}}}\,{\rm{w}}{\rm{i}}{\rm{t}}{\rm{h}}\,{D}_{i,j}={({{\boldsymbol{J}}}_{{\rm{T}},i}^{{\rm{T}}}{\boldsymbol{f}}{}_{{\rm{m}},i}({\boldsymbol{\beta }},\dot{{\boldsymbol{\beta }}},{a}_{i}))}_{j},$$

where ${D}_{i,j}$ maps the contribution of the *i-*th muscle by means of the translational Jacobian matrix ${{\boldsymbol{J}}}_{{\rm{T}},i}^{{\rm{T}}}$ to the *j*-th joint. We deployed Hill-type muscles of the form,

10 $${{\boldsymbol{f}}}_{{\rm{m}},i}({\boldsymbol{\beta }},\dot{{\boldsymbol{\beta }}},{a}_{i})={f}_{{\rm{l}}{\rm{v}},i}(s,\dot{s})\,C{}_{i}\,a{}_{i}\,{{\boldsymbol{u}}}_{i}\,{\rm{w}}{\rm{i}}{\rm{t}}{\rm{h}}\,{C}_{i}={A}_{i}\,{\sigma }_{i}\,{\rm{a}}{\rm{n}}{\rm{d}}\,i=1,...,n$$

to generate muscle forces. Therein, $n$ is the number of muscles, ${f}_{{\rm{l}}{\rm{v}},i}(\,\cdot \,)$ is the force-length-velocity relation with muscle length $s$ and muscle contraction velocity $\dot{s}$ kinematically expressed in terms of the joint angles ${\boldsymbol{\beta }}$ and joint angular velocities $\dot{{\boldsymbol{\beta }}}$, ${A}_{i}$ is the physiological cross-sectional area, ${\sigma }_{i}$ is the maximal isometric muscle stress, and ${{\boldsymbol{u}}}_{i}$ is the muscle’s unit direction vector.

For the sake of simplicity, the force-length-velocity factor was set to ${f}_{{\rm{l}}{\rm{v}},i}(\,\cdot \,)=1$ as properties of the activated muscle structures and the activation dynamics itself have little impact on the prediction of muscle forces^72^. The muscle force element is then defined as an actuator whose force generating capacity depends on its theoretical maximum force ${A}_{i}\,{\sigma }_{i}$, its activation level ${a}_{i}$, and primarily on the moment arm. Note that we used the multibody formalism for rigid bodies, as we do not expect the bone deformations to have any impact on the overall system motion or the muscle force computation.

## Supplementary information

NfMBS_2legsquat_Geier-et-al
