# Supplementary material for: Neuro-musculoskeletal flexible multibody simulation yields a framework for efficient bone failure risk assessment
Source: Sci Rep. 2019 May 6;9:6928. doi: 10.1038/s41598-019-43028-6 (PMC6503141; doi:10.1038/s41598-019-43028-6)
Supplement: Supplementary file 1 — NfMBS_2legsquat_Geier-et-al [file 41598_2019_43028_MOESM1_ESM.pdf]

Supplementary information

to the original article

Neuro-musculoskeletal flexible multibody simulation yields a framework for efficient bone failure risk assessment

Andreas Geier * [1,2], Maeruan Kebbach [1], Ehsan Soodmand [1,3],
Christoph Woernle [4], Daniel Kluess [1] and Rainer Bader [1]

[1] Department of Orthopaedics, University Medicine Rostock, Rostock, Germany
{andreas.geier, maeruan.kebbach, daniel.kluess, rainer.bader}@med.uni-rostock.de

[2] Department of Modern Mechanical Engineering, Waseda University, Tokyo, Japan
a_geier@sugano.mech.waseda.ac.jp

[3] Julius Wolff Institute for Biomechanics and Musculoskeletal Regeneration, Charité Berlin, Germany
ehsan.soodmand@charite.de

[4] Chair of Technical Dynamics, University of Rostock, Rostock, Germany
woernle@uni-rostock.de

***Correspondence and requests for materials should be addressed to**
Andreas Geier
Department of Orthopaedics, Rostock University Medical Center
Doberaner Straße 142
18057 Rostock, Germany
Tel: +49 381 498 8985
Fax: +49 381 494 9308
e-mail: andreas.geier@med.uni-rostock.de

Keywords Biomechanics, Mechanical in-vitro testing, Musculoskeletal and finite-element model, Flexible multibody dynamics, Bone stresses and strains, Bone strength, Osteoporosis, Femoral hip fracture risk

Supplementary experimental protocol of the *in-vitro* analysis. To achieve *in-vivo*-like mechanical material properties, the frozen femur was stored at room temperature (24°C) for approx. 12 h, prior to the experimental trials. The soft tissue was completely resected from the bone. Loading conditions and loading regimen are depicted in Fig. 6.

Prior to the strain gauge application, a preliminary FEA was performed to ensure proper detection of the expected principal strains. Accordingly, six uniaxial strain gauges (HBM®, Darmstadt, Germany, $\alpha = 9\text{x}10^{-6}/\text{K}$) with active temperature compensation were bonded to the specimen's superior and inferior neck as well as below to the lesser trochanter and to the middle portion, respectively, on the medial and the lateral shaft (Fig. 6.) by using a laser level ruler (Quigo Plus Cross Line Laser with Tripod, Bosch GmbH, Stuttgart, Germany). Due to internal friction during the cyclic loading, we expected sensor drift due to the change in temperature. This was compensated for by resecting a separate bone block from the femoral condyles to which an additional strain gauge was applied and therefore served as reference.

The distal end of the specimen was potted with casting resin (RENCAST FC52, Huntsman Advanced Materials, Bergkamen, Germany) into an aluminum cup with adapter flange. Likewise, the femoral head was potted via a hemisphere of casting resin into an aluminum cup with an adapter flange. To ensure torque-free mounting, the embeddings were aligned such that the force line of action went through the center of the femoral head and the midpoint of the transepicondylar axis. Moreover, the proximal adapter flange was connected via a ball bearing to the testing machine in order to eliminate shear forces.

The prepared femur specimen was mounted into a servo-hydraulic testing machine (Instron® 8872 dynamic axial-torsion system, Norwood, Massachusetts, United States) in a single leg loading configuration. Both the final bone positioning and the strain gauge alignments were verified in their final configuration using again the laser level ruler. This experimental setup should yield physiological surface strains[13]. The displacement of the force application point was measured by means of the actuator's position sensor.

The testing machine was operated in force control mode： Starting from unloaded conditions and the strain gauges reset to zero, the force was increased in ten increments from 0 N up to 1 kN with a holding time of 5 s for each of the increments. Subsequent to the static regimen, the force level was decreased from 1 kN to 550 N and a sinusoidal of the form $F\left(t\right) = 550\,\text{N} + 450\,\text{N}\,sin\left(2\omega t\right)$ with a frequency of 1 Hz and an amplitude of 450 N was applied to the bone for another 50 cycles. The complete trial was repeated n = 5 times. Aiming at the validation of the domain-specific simulation models, the experimental boundary conditions were obtained by means of a high-resolution optical 3D scan (GOM ATOS III, GOM GmbH,

Braunschweig, Germany) identifying the strain gauge locations and the fixations in the adapter flanges. More precisely, the optical 3D scanner captured the complete surface of specimen, adapter flange, and strain gauges precisely as point cloud, which was converted to a polygon mesh.

Supplementary Table SI1. Parameterization of the Hill-type muscle model.

SUPPLEMENTARY TABLE SI1
PARAMETERIZATION OF THE HILL-TYPE MUSCLE MODEL[55,73]

Muscle element	PCSA A_i [cm^2]	Volume V_i [ml]	Muscle stress σ_i [MPa]
M. flex hall long 1/1	0.001383	30	1
M. flex dig long 1/1	0.000767	25	1
M. ext hall 1/1	0.000315	16	1
M. ext dig long 1/1	0.000687	35	1
M. peron brev 1/1	0.000855	19	1
M.peron long 1/1	0.001418	42	1
M. tib ant 1/1	0.001919	75	1
M. tib post 1/1	0.00399	86	1
M. bic fem cap breve 1/1	0.000613	60	1
M. vast lat inf 1/2	0.003394	207	1
M. vast lat inf 2/2	0.003394	207	1
M. glut max inf 1/1	0.001974	316	1
M. glut med ant 1/1	0.001943	75	1
M. add brev dist 1/2	0.00009	10	1
M. add brev dist 2/2	0.00009	10	1
M. add long 1/1	0.000666	66	1
M. add magn dist 1/1	0.001778	181	1
M. glut max sup 1/1	0.001025	130	1
M. glut med post 1/1	0.00341	150	1
M. glut min ant 1/1	0.00092	26	1
M. glut min mid 1/1	0.000867	26	1
M. glut min post 1/1	0.000969	26	1
M. piriformis 1/1	0.000621	25	1
M. soleus lat 1/1	0.006698	146	1
M. soleus med 1/1	0.003924	80	1
M. vast intermed 1/1	0.001456	101	1
M. vast med sup 1/1	0.002791	246	1
M. psoas maj 1/1	0.001094	10	1
M. add magn mid 1/1	0.001074	102	1
M. add magn prox 1/1	0.000314	30	1
M. bic fem long 1/1	0.001338	111	1
M. gastrocnemius lat 1/1	0.001093	54	1
M. gastrocnemius med 1/1	0.002014	107	1
M. gracilis 1/1	0.000382	58	1
M. sartorius 1/1	0.000299	98	1
M. semimembranosus 1/1	0.001482	116	1
M. semitendinosus 1/1	0.000821	106	1
M. tensor fasciae latae 1/1	0.000395	34	1
M. rectus fem 1/2	0.00078	107	1
M. rectus fem 2/2	0.00078	107	1

Supplementary information on the validation of the musculoskeletal multibody model.

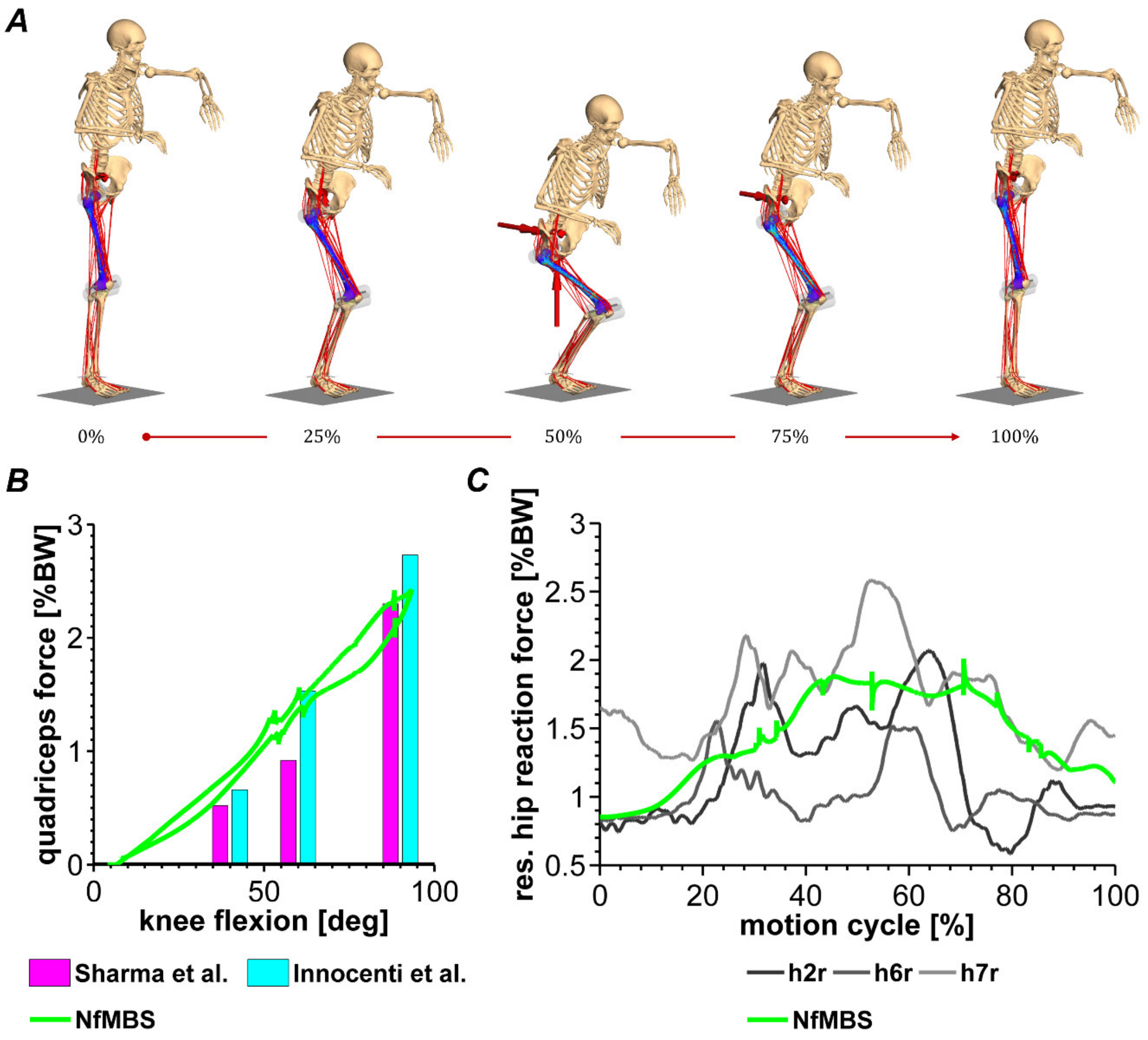

Figure SI2. Validation of the musculoskeletal multibody model with the flexible femur superelement. The boundary conditions due to musculoskeletal dynamics during a squat motion (A) have been validated in terms of the quadriceps force (B) and the resultant hip reaction force (C). The quadriceps force captures the direct (due to insertion at the bone) and indirect (due to spanning the knee and hip joint) effects of the muscle forces on the femur and is compared against two simulation studies: Sharma *et al.* [57] and Innocenti *et al.* [58]. The resultant reaction force in the hip joint is an important validation parameter for joint dynamics: it is compared against the *in vivo* measurements of instrumented hip replacements for three subjects (h2r, h6r, and h7r) as described in Bergmann *et al.* [59].

References

73. An, K.N., Kaufman, K.R. & Chao, E.Y. Physiological considerations of muscle force through the elbow joint. *Journal of biomechanics*. **22,** 1249–1256 (1989).

Supplementary media files

Description. Online supplementary files for the publication 'Neuro-musculoskeletal flexible multibody simulation yields a framework for efficient bone failure risk assessment'. The *Supplementary material* contains a demonstration video showing the dynamic stresses that occur during a two-leg squat as captured in the gait lab. **Packing list.** NfMBS_2legsquat_Geier-et-al.mpg.
